# Prescription drugs with potential for misuse in Irish prisons: analysis of national prison prescribing trends, by gender and history of opioid use disorder, 2012 to 2020

**DOI:** 10.1186/s12888-023-05195-9

**Published:** 2023-10-06

**Authors:** Louise Durand, Eamon Keenan, Deirdre O’Reilly, Kathleen Bennett, Andy O’Hara, Gráinne Cousins

**Affiliations:** 1 School of Pharmacy and Biomolecular Sciences, RCSI University of Medicine and Health Sciences, 123 St. Stephen’s Green, Dublin 2, Ireland; 2 National Social Inclusion Office, Health Service Executive, Mill Lane, Palmerstown, Dublin 20, Ireland; 3 Irish Prison Service, IDA Business Park, Ballinalee Road, Longford, Ireland; 4 Data Science Centre, School of Population Health, RCSI University of Medicine and Health Sciences, Lower Mercer Street, Dublin 2, Ireland; 5 UISCE, National Advocacy Service for People who use Drugs in Ireland, 8 Cabra road, Dublin 7, Ireland

**Keywords:** Prison prescribing, Prescription opioids, Benzodiazepines, Z-drugs, Gabapentinoids, Opioid use disorder, Gender

## Abstract

**Background:**

Pharmacotherapy is essential for the delivery of an equivalent standard of care in prison. Prescribing can be challenging due to the complex health needs of prisoners and the risk of misuse of prescription drugs. This study examines prescribing trends for drugs with potential for misuse (opioids, benzodiazepines, Z-drugs, and gabapentinoids) in Irish prisons and whether trends vary by gender and history of opioid use disorder (OUD).

**Methods:**

A repeated cross-sectional study between 2012 and 2020 using electronic prescribing records from the Irish Prison Services, covering all prisons in the Republic of Ireland was carried out. Prescribing rates per 1,000 prison population were calculated. Negative binomial (presenting adjusted rate ratios (ARR) per year and 95% confidence intervals) and joinpoint regressions were used to estimate time trends adjusting for gender, and for gender specific analyses of prescribing trends over time by history of OUD.

**Results:**

A total of 10,371 individuals were prescribed opioid agonist treatment (OAT), opioids, benzodiazepines, Z-drugs or gabapentinoids during study period. History of OUD was higher in women, with a median rate of 597 per 1,000 female prisoners, compared to 161 per 1,000 male prisoners. Prescribing time trends, adjusted for gender, showed prescribing rates decreased over time for prescription opioids (ARR 0.82, 95% CI 0.80–0.85), benzodiazepines (ARR 0.99, 95% CI 0.98–0.999), Z-drugs (ARR 0.90, 95% CI 0.88–0.92), but increased for gabapentinoids (ARR 1.07, 95% CI 1.05–1.08). However, prescribing rates declined for each drug class between 2019 and 2020. Women were significantly more likely to be prescribed benzodiazepines, Z-drugs and gabapentinoids relative to men. Gender-specific analyses found that men with OUD, relative to men without, were more likely to be prescribed benzodiazepines (ARR 1.49, 95% CI 1.41–1.58), Z-drugs (ARR 10.09, 95% CI 9.0-11.31), gabapentinoids (ARR 2.81, 95% CI 2.66–2.97). For women, history of OUD was associated with reduced gabapentinoid prescribing (ARR 0.33, 95% CI 0.28–0.39).

**Conclusions:**

While the observed reductions in prescription opioid, benzodiazepine and Z-drug prescribing is consistent with guidance for safe prescribing in prisons, the increase in gabapentinoid (primarily pregabalin) prescribing and the high level of prescribing to women is concerning. Our findings suggest targeted interventions may be needed to address prescribing in women, and men with a history of OUD.

**Supplementary Information:**

The online version contains supplementary material available at 10.1186/s12888-023-05195-9.

## Introduction

Misuse, or nonmedical use of prescription drugs refers to the intentional repurposing of prescribed drugs outside of their intended indication, or to the use of illicitly sourced prescription drugs [1–3]. Drugs identified with the greatest potential for misuse are prescription opioids, benzodiazepines, Z-drugs and gabapentinoids [1, 4, 5]. The International Narcotics Control Board warned, over a decade ago, that the misuse of these prescription drugs could exceed illicit drug use [6]. The majority of research describing this phenomenon has come from the United States where the widespread availability of prescription opioids has driven the opioid epidemic in North America [4]. While much of the attention has been devoted to the misuse of prescription opioids, the United Nations Office on Drugs and Crime (UNODC) indicated in 2017 that polydrug use, particularly with benzodiazepines, may be linked to the increase in prescription opioid deaths and that the misuse of benzodiazepines is a growing public health threat [7]. In fact, the UNODC 2018 Report identified benzodiazepines as one of the most commonly misused prescription drugs, with approximately 60 countries ranking benzodiazepines among the three most commonly misused substances [8]. Benzodiazepines are indicated for short-term treatment of severe anxiety and insomnia (< 4 weeks) [9]. While benzodiazepines have a relatively low toxicity profile, concurrent use with opioids increases the risk of overdose, arising from cumulative and synergistic effects on respiratory depression [10]. Z-drugs, a comparatively newer group of non-benzodiazepine hypnotic agents, licensed for use in the 1990s for insomnia, have a similar risk profile to benzodiazepines [11, 12]. Gabapentinoids (the γ-aminobutyricacid [GABA] analogue medications pregabalin and gabapentin) are licensed for the treatment of epilepsy, neuropathic pain, and generalised anxiety disorder (pregabalin only). Evidence from recent systematic reviews demonstrates that gabapentinoids, although originally classified as having low misuse potential, are increasingly misused to achieve euphoria, sedation, or dissociation. Furthermore, they are frequently used with other drugs, with opioid use disorder the greatest risk factor for misuse of gabapentinoids, particularly pregabalin [13, 14]. Similar to the effects of benzodiazepines, when taken with opioids, gabapentinoids may cause a dangerous respiratory depression resulting in mortality [15].

Prisons have been identified as a high risk setting for the misuse and harm associated with both prescription and illicit drugs, with an estimated 30% of men and 51% of women in prisons worldwide identified as having a drug use disorder [16]. Most recent estimates in Ireland suggest a higher prevalence, with approximately 51% of men and 63% of women in Irish prisons identified as having a substance use disorder [17]. While pharmacotherapy and prescribing of medications is essential for the delivery of an effective and equivalent standard of care in prisons, prescribing can be very challenging due to the complex health needs of prisoners and the risks to the individual and wider prison population associated with the misuse and diversion of prescription drugs and other illicit substances [18–21].

In response to increasing concerns regarding the misuse of prescription drugs in prisons, the UK Royal College of General Practitioners published updated guidance for clinicians on safe prescribing in the prison setting in 2019 [22]. The guidance uses a traffic light system, discouraging clinicians from prescribing ‘red medicines’ to carefully considering ‘amber medicines’ and choosing ‘green medicines’ as first choice. For example, benzodiazepines are classified as ‘red medicines’ and are not recommended for the treatment of insomnia or anxiety in prison. It is recommended that the prescribing of benzodiazepines in prison should be primarily limited to use in assisted withdrawal (detoxification) from benzodiazepine and alcohol dependence. Similarly, pregabalin and gabapentin are coded red and not recommended for treatment of anxiety, neuropathic pain or epilepsy in prison due to high risk of misuse and diversion. Z-drugs are classified as amber or second-line treatment for insomnia, but caution is advised due to risk of diversion and illicit use. Methadone is recommended as the first-line Opioid Agonist Treatment (OAT) unless the prisoner is already stabilised on buprenorphine [22].

Although there is widespread concern regarding the misuse of prescription drugs in prisons, few studies have examined prescribing trends of prescription drugs with potential for misuse in prison. One cross-sectional study, using a census day methodology, found a high prevalence of psychotropic prescribing across 11 prisons in England, with approximately 17% of men and 48% of women prescribed at least one psychotropic medicine. Women were eight times more likely than men to be prescribed hypnotic and anxiolytic medicines [18]. Furthermore, despite the significant burden of opioid use in prisons [23], it remains unknown if prescribing practices for prescription opioids, benzodiazepines, Z-drugs and gabapentinoids in prison vary depending on whether a person has a history of opioid use disorder (OUD) or is in receipt of OAT. Evidence from studies in the community suggests high levels of co-prescribing for prescription opioids, benzodiazepines, Z-drugs, and gabapentinoids to patients in receipt of OAT [24–28], posing an elevated risk of overdose mortality [29, 30].

The Irish Prison Service is responsible for all 12 prisons in the Republic of Ireland, with a total operational capacity of 4,375 people (4,201 men and 174 women) [31]. The prison healthcare service provides all people in prison with access to free healthcare services, including prescription medications. Opioid agonist treatment is available in 10 of the 12 prisons (accommodating over 80% of the prison population). Any person entering prison with a history of opioid use and testing positive for opioids is offered a medically assisted detoxification, if clinically indicated. People in prison who, on committal, are engaged in OAT in the community, have their substitution treatment continued while in prison [32].

This study aims to (1) examine prescribing rates and trends for opioids, benzodiazepines, Z-drugs, and gabapentinoids in Irish Prisons between 2012 and 2020 using electronic health records data from the Irish Prison Services; (2) Examine whether prescribing rates and trends vary by gender and if a person has a history of OUD; (3) Determine rates of co-prescribing of opioids, benzodiazepines, Z-drugs, or gabapentinoids among people in receipt of OAT medications.

## Methods

The protocol relating to this study was published elsewhere [33]. The study is reported according to the Strengthening the Reporting of Observational Studies in Epidemiology (STROBE) guidelines, REporting of studies Conducted using Observational Routinely-collected health Data (RECORD) statement [34, 35].

### Design

This is a repeated cross-sectional study using anonymised individual-level prescribing data obtained from the Irish Prison Services, from all prisons in Ireland between March 2012 and December 2020.

### Participants and settings

The Irish Prison Service uses a centralised electronic patient record (Prisoner Healthcare Management System), which includes records of medications prescribed to people while in prison in Ireland as well as gender, month of birth, prison sentence (first, second, etc.), and start and end dates of each sentence. Data was included on all individuals who were prescribed opioids, benzodiazepines, Z-drugs, gabapentinoids, or OAT (methadone or buprenorphine) for OUD at least once while in prison in Ireland during the observation period. Medications of interest were coded using the WHO Anatomical Therapeutic Chemical (ATC) classification System [36]. Table 1 provides an overview of the drugs of interest. We excluded 3 prisoners with missing data on year of birth. If a prescription was running further than the recorded release date, we used the release date as the prescription end date.

**Table 1:** Drugs included in the study by drug class and ATC code

Drug class	ATC code	ATC level name
Benzodiazepines	N03AE01	clonazepam
	N05BA01	diazepam
	N05BA02	chlordiazepoxide
	N05BA06	lorazepam
	N05BA08	bromazepam
	N05BA09	clobazam
	N05BA12	alprazolam
	N05CD01	flurazepam
	N05CD02	nitrazepam
	N05CD05	triazolam
	N05CD06	lormetazepam
	N05CD07	temazepam
	N05CD08	midazolam
Z-drugs	N05CF01	zopiclone
	N05CF02	zolpidem
	N05CF03	zaleplon
Gabapentinoids	N03AX12	gabapentin
	N03AX16	pregabalin
Prescription Opioids	N02AA01	morphine
	N02AA05	oxycodone
	N02AA51	morphine, combinations
	N02AA55	oxycodone and naloxone
	N02AB03	fentanyl
	N02AE01	buprenorphine
	N02AJ13	tramadol and paracetamol
	N02AX02	tramadol
	N02AX05	meptazinol
	N02AX06	tapentadol
Opioid agonist treatment^a^	N07BC01	buprenorphine
	N07BC02	methadone
	N07BC51	buprenorphine, combinations

^a^Used to identify a history of opioid use disorder

### Data and outcomes

We calculated monthly prescribing rates per 1,000 prison population in Ireland for each drug class of interest, and by individual ATC code, between March 2012 to December 2020. We used the number of people prescribed medication at least once during the month as the numerator and the total number in prison on the last day of each month as the denominator. Repeat prescriptions were counted each month where dispensing occurred. Annual average prescribing rates were also calculated as the mean of monthly rates observed each year.

History of OUD was defined, as ever receiving a prescription for OAT (N07BC01, N07BC02 or N07BC51) while in prison during the study period. While some people with OUD may not engage in treatment, prison policy in Ireland recommends that OAT (detoxification or maintenance) is offered to all prisoners with OUD. Co-prescribing to people in receipt of OAT, was identified as having a prescription for methadone or buprenorphine (N07BC01, N07BC02 or N07BC51) with a concurrent prescription of  ≥ 7 days during a given month for an opioid, benzodiazepine, Z-drug or gabapentinoid. Co-prescribing rates were estimated per 1,000 prison population with a history of OUD on the last day of each month. Annual average co-prescribing rates were also calculated as the mean of monthly co-prescribing rates observed each year.

### Statistical analysis

#### Descriptive analysis

Men and women in prison with at least one prescription for opioids, benzodiazepines, Z-drugs, gabapentinoids, or OAT medication were compared, based on their demographic and sentence characteristics. Reporting means and standard deviation by gender, t-tests were used to evaluate gender differences. Median and quartiles of total number of days covered in those prescribed are reported by drug class. Median and quartiles history of OUD rates are presented by gender.

#### Prescribing trends

Prescribing rates for opioids, benzodiazepines, Z-drugs, and gabapentinoids are reported as annual average rates (overall, and by gender). Time trends in prescribing rates, adjusting for gender, were examined using a negative binomial regression. We report adjusted rate ratios (ARR) with 95% confidence intervals (CI).

#### Gender and history of OUD

Given the anticipated gender differences in prescribing rates, as well as small group size, potentially resulting in instability of model estimates, we used separate negative binomial regression models to estimate prescribing trends, adjusting for history of OUD, for men and women. The log of the population size was included as an offset term. Graphs and ARR with 95% CI for time (per year) and history of OUD are reported. Where our analyses of crude trends suggested inflection points, joinpoint regression [37] was used to formally identify change points in trends, reporting the annual percent changes (APC). Joinpoint regression analyses were stratified by gender and history of OUD, using uncorrelated, constant error variance assumptions and permutation test model selection method [38].

#### Co-prescribing with OAT

Annual average co-prescribing rates for OAT medications with opioids, benzodiazepines, Z-drugs, or gabapentinoids are presented graphically by gender. Median and quartiles of monthly co-prescribing rates are reported. Time trends are assessed using negative binomial regression of monthly co-prescribing rates, reporting unadjusted rate ratios.

SA﻿S Enterprise Guide (v 7.1) [39] and Joinpoint regression program [37] were used for analyses and significance at *p* < 0.05 were assumed.

## Results

 The number of people incarcerated in Irish prisons ranged from 4,340 (March 31st 2012) to 3,650 (December 31st 2020). Women represented approximately 3.8% of the prison population during the study period. Characteristics of the individuals (*N* = 10,371) who were prescribed at least one opioid, benzodiazepine, Z-drug, gabapentinoid or OAT medication while in prison between March 2012 and December 2020 are presented in Table 2 ; 15.7% (*n* = 1,628) of them were women, 41.4% were aged ≥ 35 years on their first committal (median 32.9 years) and 42.8% (*n* = 4,439) were prescribed OAT medication only. The median total time spent in prison was 43.6 months [IQR 5–269]. Women experienced significantly fewer prison sentences, with an average of 5 sentences (SD 5.6), compared to a male average of 6 (SD 5.4) (t-test *p* < 0.01). Women also served shorter sentences. OUD was higher among women in prison with a median [IQR] rate of 597 [544–631] per 1,000 female prisoners in receipt of OAT vs. 161 [150–167] per 1,000 male prisoners.

**Table 2:** Characteristics of study population^a^ (all data relates to the period 2012 to 2020)

		Men (*N* = 8,743)	Women (*N* = 1,628)	Total (*N* = 10,371)	*P*-value^b^
Age (years) at first sentence	Mean (SD)	34.5 (9.9)	33.8 (9.5)	34.4 (9.9)	< 0.01
Total number of sentences		6.3 (5.4)	5.4 (5.6)	6.2 (5.4)	< 0.01
Duration of the longest sentence (months)		18.4 (25.4)	9.1 (15.2)	17.0 (24.3)	< 0.01
Total days prescribed benzodiazepines or Z-drugs [among those prescribed benzodiazepines or Z-drugs, *N* = 4701]	Median [IQR]	27 [12–54]	24 [7–59]	27 [10–54]	
Total days prescribed gabapentinoids [among those prescribed gabapentinoids, *N* = 1405]	Median [IQR]	119 [21–504]	18.5 [6–73]	97 [16–432]	
Total days prescribed opioids [among those prescribed opioids, *N* = 1556]	Median [IQR]	19 [7–105]	18 [5–112.5]	19 [7–105.5]	

^a^Population includes only those individuals who were prescribed opioids, benzodiazepines, Z-drugs, gabapentinoids, or opioid agonist treatment (methadone or buprenorphine) for OUD at least once while in prison in Ireland during the observation period

^b^Student-t test comparing genders

### Prescribing trends

**Fig. 1:**
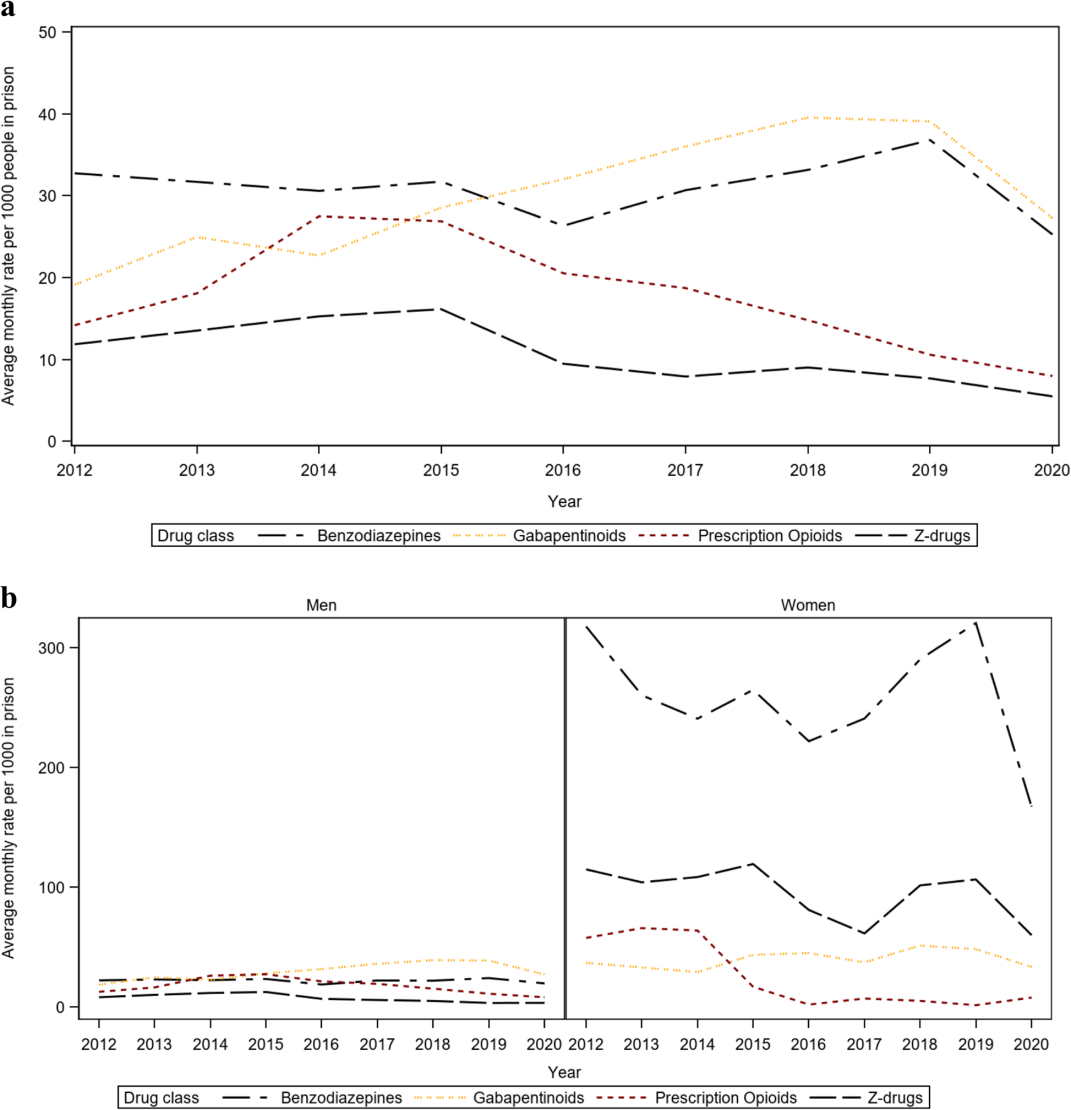
Annual average prescribing rates for opioids, benzodiazepines, Z-drugs and gabapentinoids in prison between 2012 and 2020, overall (**a**) and by gender (**b**)

Annual average prescribing rates for opioids, benzodiazepines, Z-drugs, and gabapentinoids in prison are presented in Fig. 1, overall (1a) and by gender (1b).

After adjusting for time trends, women were significantly more likely to be prescribed benzodiazepines (ARR  [95% CI]  11.81 [11.17–12.48]), Z-drugs (ARR [95% CI] 13.61 [12.28–15.08]) and gabapentinoids (ARR  [95% CI]  1.35 [1.23–1.48]) while in prison relative to men. Because of the high occurrence of null rates for prescription opioids in the second half of the study period in women, we do not report gender comparisons for this drug class. Adjusted time trends across genders show prescribing rates were decreasing over the study period for benzodiazepines (ARR [95% CI] 0.99 [0.98–0.999]), Z-drugs (ARR [95% CI] 0.9 [0.88–0.92]) and prescription opioids (ARR [95% CI] 0.82 [0.8–0.85]), whereas they were increasing for gabapentinoids (ARR [95% CI] 1.07 [1.05–1.08]). We also note a decline in prescribing rates of all drugs classes of interest in 2020 compared with 2019. A reduction in prescribing of OAT medication also occurs in 2020 (-22% vs. 2019).

Annual average prescribing rates are presented by individual drug (ATC code) and by gender in the additional files (Additional figure). Chlordiazepoxide and diazepam were the most frequently prescribed benzodiazepines in prison, with other benzodiazepines showing a considerably lower occurrence. Among gabapentinoids, pregabalin prescribing rates were higher than gabapentin in both men and women. Tramadol and the combination of tramadol and paracetamol were the most common prescription opioids. We also note that in women, opioid prescribing was consistently very low (close to zero) after 2015.

### Prescribing trends by gender and history of OUD

**Fig. 2:**
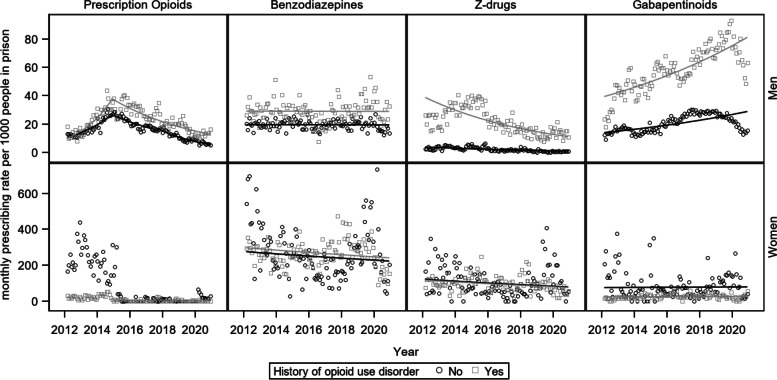
Monthly prescribing rates of benzodiazepines, Z-drugs, prescription opioids and gabapentinoids in prison by gender and history of opioid use disorder – the Y-axis scale differs between genders

Figure 2 presents the observed and predicted monthly prescribing rates for each drug class, by history of OUD, stratified by gender. Adjusted rate ratios for history of OUD and time are presented separately for each drug class and by gender in Table 3. Men with a history of OUD were more likely to be prescribed benzodiazepines (ARR [95% CI] 1.49 [1.41–1.58]), Z-drugs (ARR [95% CI] 10.09 [9–11.31]), and gabapentinoids (ARR [95% CI] 2.81 [2.66–2.97]) than men without OUD. An increasing trend was observed in gabapentinoid (ARR [95% CI]  1.09 [1.07–1.1]) prescribing rates whereas Z-drugs significantly decreased over the study period (ARR [95% CI] 0.87 [0.85–0.89]). Opioid prescribing rates remained higher in men with a history of OUD than those without throughout the study period. Joinpoint regression results are presented in Table 4. One inflection point was identified in men with a history of OUD, and two inflection points in men without a history of OUD. In men with a history of OUD, opioid prescribing rates were increasing sharply (APC + 62%) until December 2014, reaching 35 per 1,000 in prison. Subsequently, the rates decreased until the end of the study period with an annual percent change of -16.5%. A similar dynamic was observed among men without a history of OUD, with opioid prescribing rates increasing (APC 40%) until December 2014, and decreasing thereafter (APC Dec 14 to Mar 18 -17.3% and Mar 18 to Dec 20 -28.6%).

**Table 3:** Adjusted rate ratios [95% CI] associated with history of opioid use disorder (OUD) and time trends for the prescription of benzodiazepines, Z-drugs and gabapentinoids, for women and men in prison

		Adjusted rate ratios [95%CI]	
Drug class	Parameter	Men	Women
Benzodiazepines	History of OUD	1.49 [1.41–1.58]	1.08 [0.97–1.2]
	time (per year)	1 [0.99–1.01]	0.98 [0.96–0.996]
Z-drugs	History of OUD	10.09 [9–11.31]	0.97 [0.83–1.13]
	time (per year)	0.87 [0.85–0.89]	0.95 [0.93–0.98]
Gabapentinoids	History of OUD	2.81 [2.66–2.97]	0.33 [0.28–0.39]
	time (per year)	1.09 [1.07–1.1]	1.01 [0.97–1.04]

**Table 4:** Annual percent change and confidence interval identified between specific change points for the opioid prescription rates in men in prison with and without history of opioid use disorder (OUD)

History of OUD (Men only)	Lower Endpoint	Upper Endpoint	Annual percent change [95%CI]
No	Mar-12	Dec-14	40 [30.2–50.4]
	Dec-14	Mar-18	-17.3 [-23.2 - -11]
	Mar-18	Dec-20	-28.6 [-33.5 - -23.2]
Yes	Mar-12	Dec-14	62 [45.9–79.9]
	Dec-14	Dec-20	-16.5 [-18.8 - -14.1]

In contrast, for women, having a history of OUD was associated with lower gabapentinoid (ARR [95%CI] 0.33 [0.28–0.39]) prescribing, while no significant difference was observed in benzodiazepine and Z-drug prescribing rates. Decreasing time trends were found in Z-drug and benzodiazepine prescribing rates with a 5% and 2% reduction, respectively, per year.

### Co-prescribing with OAT

**Fig. 3:**
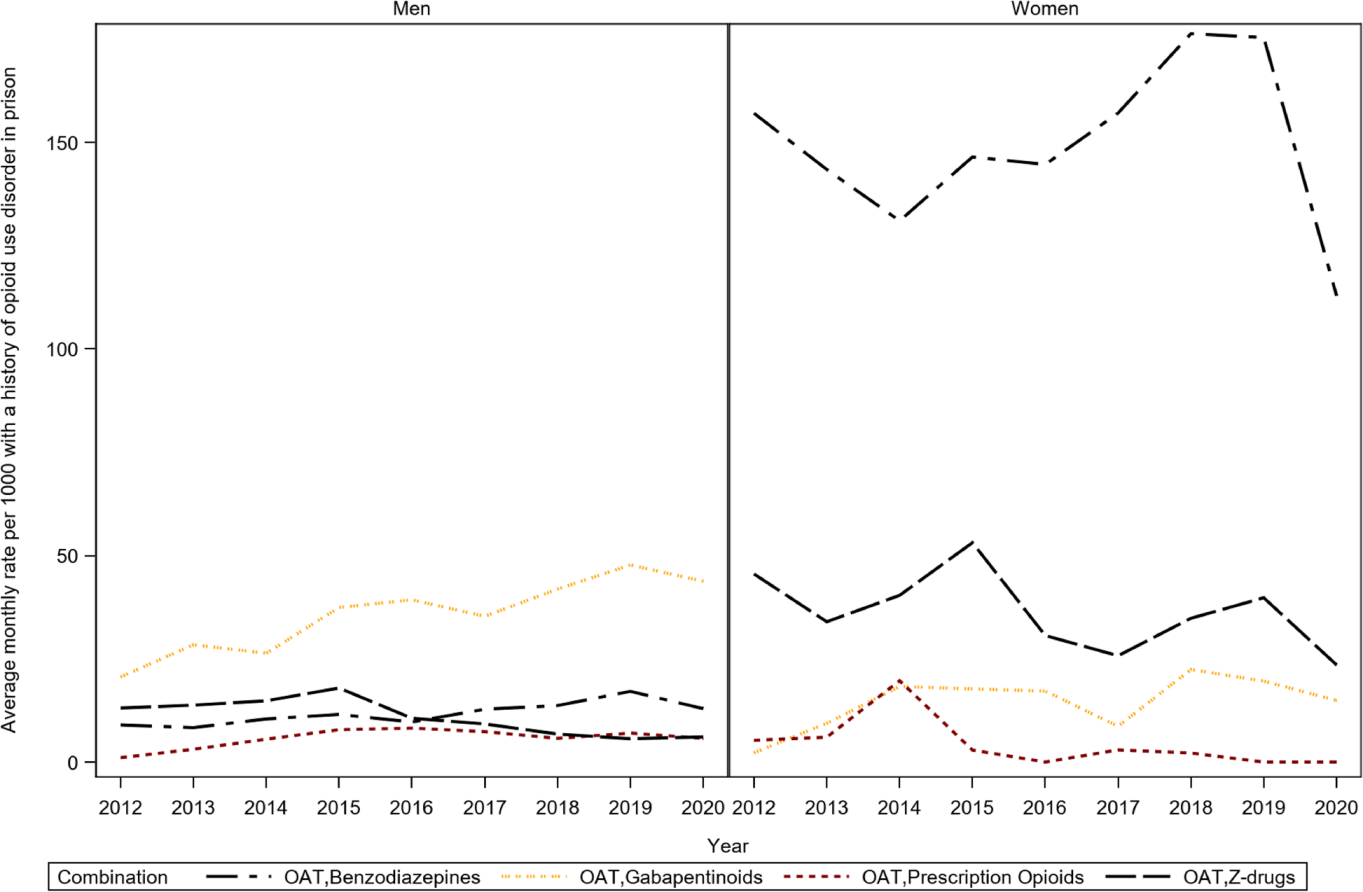
Annual average prescribing rates of OAT in combination with opioids, benzodiazepines, Z-drugs and gabapentinoids

Methadone was the most commonly prescribed OAT in prisons, with monthly prescribing rates ranging from (min-max) 364–723 per 1000 women and 96–163 per 1000 men. This compares to monthly prescribing rates of (min-max) 0 to 13 per 1000 women for buprenorphine/ naloxone combination, and 0 to 1.3 per 1000 men. Figure 3 shows the annual average co-prescribing rates for OAT medications with opioids, benzodiazepines, Z-drugs, and gabapentinoids among men and women. Median and quartiles of monthly co-prescribing rates are reported in Table 5. The highest level of co-prescribing with OAT drugs is found for benzodiazepines in women, with a median of 150 [IQR 122–180] per 1000 women with a history of OUD. Co-prescribing of OAT and other opioids was low with a median of 6 [IQR 4–8] per 1000 men and 0 [IQR 0–8] for women with a history of OUD. Gabapentinoids were the most co-prescribed medications in men with a median of 38 [IQR 28–43] per 1000 with history of OUD, compared to 13 [IQR 0–23] per 1000 women with history of OUD.

**Table 5:** Median and quartiles of co-prescribing rates per 1000 with a history of opioid use disorder; Rate ratios and 95% confidence intervals for time trends (per year) for co-prescribing of OAT and opioids, benzodiazepines, Z-drugs and gabapentinoids

	Median [Q1 – Q3] prescribing rate		Rate Ratio [95% CI]	
Combination	Men	Women	Men	Women
OAT,Prescription opioids	6 [4–8]	0 [0–8]	1.11 [1.07–1.15]	N/C
OAT,Benzodiazepines	12 [8–15]	150 [122–180]	1.08 [1.05–1.11]	1.01 [0.98–1.03]
OAT,Z-drugs	10 [7–14]	33 [19–50]	0.88 [0.86–0.91]	0.95 [0.91–1.00]
OAT,Gabapentinoids	38 [28–43]	13 [0–23]	1.09 [1.08–1.11]	1.1 [1.04–1.17]

Unadjusted rate ratio estimates for time trends are reported in Table 5. Co-prescribing rates increased for gabapentinoids (RR [95% CI]  1.1 [1.04–1.17]) in women. Co-prescribing rates increased among men for gabapentinoids (RR [95% CI] 1.09 [1.08–1.11]), benzodiazepines (RR [95% CI] 1.08 [1.05–1.11]), and, opioids (RR [95% CI] 1.08 [1.08–1.11]), but decreased for Z-drugs (RR [95% CI] 0.89 [0.87–0.92]).

## Discussion

### Summary of results in context of previous research

In this study, we examined trends in the prescribing of opioids, benzodiazepines, Z-drugs, and gabapentinoids in Irish Prisons between 2012 and 2020. We identified an overall significant reduction over time in prescribing of opioids (since 2015), benzodiazepines and Z-drugs, and an increase in gabapentinoid prescribing. The prison population is younger and has disproportionately more men compared to available evidence from the national pharmacy claims database in Ireland, which renders comparisons difficult. Nevertheless, observations of long-term prescribing trends in the community identified an increase in prescription opioids from 2010 to 2019 [40] and Z-drugs between 2005 and 2015 [41], which are not reflected in our prison population. Observations of decreasing benzodiazepines between 2005 and 2015 [41], and increasing pregabalin prescribing between 2013 and 2016 [42] in the community are consistent with trends observed in our prison population.

It is also worth noting that, compared to the community [40, 41], a limited number of benzodiazepines and opioids were consistently prescribed in prison. Chlordiazepoxide and diazepam were the benzodiazepines of choice in prisons, whereas diazepam, alprazolam and temazepam were identified as the most commonly dispensed benzodiazepines in a previous study using pharmacy dispensing records in the community [41]. The high rate of prescribing for chlordiazepoxide may be due to the high prevalence of alcohol use disorders among prisoners [16, 17]. Chlordiazepoxide is recommended for uncomplicated alcohol withdrawal as it has a low dependence-forming potential [22, 43], whereas diazepam is recommended if there is a history of concurrent benzodiazepine dependence [22]. Opioid prescriptions in prisons were almost entirely limited to tramadol, similar to findings from a study in Swiss prisons [44]. A repeated cross-sectional analysis of the national community pharmacy claims database in Ireland between 2010 and 2019 also identified tramadol as the most frequently prescribed product, however oxycodone and tapentadol prescribing were increasing over the study period [40].

The Irish prison population was largely composed of men (96%), similar to other EU countries, where women generally represent only 3 to 8% of the prison population [45]. Although women represented less than 5% of the Irish prison population, they had a high burden of OUD. While a recent meta-analysis of 24 prison studies also identified a higher pooled prevalence estimate of OUD among women (51%) compared to men (30%), it would appear that OUD among women in Irish prisons (60%) is high compared to international studies, but low for men (16%) in Irish prisons [16]. This is also in contrast to recent estimates in the Irish community where the prevalence of problematic opioid use among men (10 per 1,000) is over twice that of women (4 per 1,000) [46].

Prescribing rates of benzodiazepines and Z-drugs in prison were almost 12 and 14 times higher, respectively, in women compared to men. These estimates are comparable to recent UK results which reported an 8-fold increase in prescriptions for hypnotics and anxiolytics to women relative to male prisoners [18]. While women in Irish prisons were also more likely to be prescribed gabapentinoids compared to men, the difference was of a lesser magnitude.

A history of OUD affected men and women prescribing rates differently. In men, a history of OUD was associated with significantly higher rates of prescribing for opioids, benzodiazepines, z-drugs, and gabapentinoids. By contrast, women with a history of OUD were less likely than other women to be prescribed gabapentinoids, and no difference was observed for benzodiazepines and Z-drugs. Co-prescribing of opioids, benzodiazepines, Z-drugs or gabapentinoids with OAT drugs remained uncommon throughout the study period, with the exception of benzodiazepines in women. Co-prescribing benzodiazepines, Z-drugs or gabapentinoids with OAT is identified as a risk factor for drug related mortality [30]. A prior study in specialist addiction clinic settings in Ireland identified up to 65% of OAT clients with a history of co-prescribing of OAT and benzodiazepines between 2010 and 2015 [24]. In this regard, co-prescribing rates appear conservative in prison settings, reducing the risk of drug poisoning mortality in prisoners on OAT. It is plausible that a higher proportion of people receive short-term detoxification in prisons [47] compared to community and specialist addiction clinic services, which can explain, in part, the lower co-prescribing rates found here.

In 2020, we observed a marked decrease in prescribing of benzodiazepines and gabapentinoids, contrasting with previous years (Fig. 1a). COVID-19 public health measures introduced in the first quarter of 2020 resulted in a reduction of court activity and delays in the justice system, with a decrease in the number of committals, particularly for short sentences (< 3 months) [31]. In addition, contingency measures were introduced to mitigate the effects of COVID-19 in people who use drugs and ensure continuity of treatment for people on OAT. These included accelerated access to OAT for people not already in treatment, additional emergency accommodation in COVID-19 facilities to allow for self-isolation/ social distancing among homeless people [48]. This may have reduced the number of committals of people with a history of OUD, particularly the more complex cases, and in turn reduced the observed prescribing rates.

### Clinical implications

In several respects, prescribing practices in Irish prisons appear to adhere to the UK Royal College of General Practitioners’ guidelines for safe prescribing in prisons [22]. Firstly, methadone was the OAT drug of choice, and, with the exception of benzodiazepines in women, the levels of co-prescribing of OAT with other opioids, benzodiazepines, Z-drugs or gabapentinoids remained very low. Secondly, the reduction in prescribing for Z-drugs, benzodiazepines and opioids may reflect an increased awareness of the potential for misuse of these drugs in prison settings. In addition, the sharp decline in opioid prescriptions from 2015 may be in response to emerging evidence from the US opioid crisis which identified an increase in opioid overdose deaths arising from poor prescribing practices, with synthetic opioids such as tramadol increasingly implicated in drug poisonings in the US from 2015 [49]. It also coincides with tramadol being classified as a Schedule 3 controlled substance in June 2014 in the U.K, after concerns about safety and potential risk of misuse were raised [50]. In contrast, gabapentinoid prescribing increased during the observation period despite recommendations to avoid in prison [22]. It does, however, appear to be reducing in 2020, following recent advice regarding appropriate prescribing of pregabalin issued by the Health Service Executive in Ireland in 2019 to all general practitioners [42]. Nevertheless, given the risk of dependence and the potential for diversion and medicinal misuse of pregabalin, prescribing trends should be monitored, to determine whether the downturn observed in 2020 continues, or rebounds to pre-pandemic levels. The sharp reduction observed in opioid prescribing since 2015 and increase in gabapentinoids should also prompt an examination of pain management practices. Acknowledging it is challenging for prescribers to balance the risk of misuse of strong analgesic medications in prison settings [44], prisoners with untreated chronic pain may seek illicitly sourced analgesic drugs, increasing the risk of adverse effects, including dependence and mortality.

Findings from this study add to existing evidence on prescribing practices in prisons but also provide new evidence in relation to how prescribing practices vary by gender and history of OUD. Women were more often prescribed benzodiazepines, Z-drugs and gabapentinoids than men, and men with a history of OUD were more often prescribed benzodiazepines, Z-drugs and gabapentinoids compared to other men. While this may reflect the more complex needs of women and people with a history of OUD, including increased levels of pain [51] and mental health issues [17, 52], further work is needed to understand if other factors are driving these differences. As previously noted, additional targeted social and psychological support could be beneficial, to reduce the reliance on benzodiazepines and Z-drugs prescribing, when clinically appropriate [18]. In line with existing evidence, we found a high prevalence of OUD in Irish prisons. Prison represents an opportunity to engage people in addressing health issues including OUD, in this otherwise difficult to reach population [53]. Easy access to services and improved adherence to OAT within the prison environment can be viewed as favourable conditions to initiate maintenance treatment, if OAT can be secured seamlessly in the community after release [54, 55].

Due to the limited number of prisons and prescribers involved, a change of prescribers and/or prescribing practices in a single prison can greatly affect national estimates. While this can be seen as a challenge to standardisation and continuity of care, it can also provide opportunities for rapid change and implementation of recommended prescribing practices.

### Strengths and limitations

This study is not without limitations. Firstly, we used the number of people in prison on the last day of the month as the denominator to estimate monthly prescribing rates, with the number of prisoners prescribed at any time during the month as the numerator. This results in an overestimate of monthly prescribing rates. However, the overestimation is expected to be consistent throughout the study period, therefore providing an accurate evaluation of trends. In addition, people with a history of OUD who did not receive a prescription for OAT during the study period will be misclassified as not having a history of OUD. This could affect the external validity of the study, however, as all people with OUD in prison should be offered treatment (detoxification or maintenance), misclassification is expected to remain low. Secondly, due to insufficient numbers in certain subgroups, we limited adjustment factors to gender and history of OUD. Residual confounding cannot be excluded from the trend analysis. Thirdly, prescription records did not contain information on diagnosis or indication for prescribing, or dosage or duration of treatment, therefore limiting our ability to assess appropriateness of prescribing against existing guidelines. Fourthly, we selected a minimum of 7-days overlap per month, as a meaningful indication of co-prescribing among people in receipt of OAT in prison. While aiming to exclude once-off prescriptions for acute situations, our cut-off misclassifies co-prescriptions of 1–6 days per month as none. Thus, co-prescribing rates are underestimated. Fifthly, while all drugs analysed in this study would have been taken under supervision in the prison, we do not know to what extent doses were concealed within the mouth and later removed and diverted to other prisoners, either voluntarily or under duress. Sixthly, benzodiazepine prescribing rates did not include Prazepam (N05BA11), as it was not retrieved from the prescription records. However, considering the relatively low prescribing rates in the community [41], and the predominance of diazepam and chlordiazepoxide in prison, we expect this had little impact on the overall prescribing trends for benzodiazepines in this study. Finally, our analysis did not include sedating anti-depressants (e.g. mirtazapine) or antipsychotics (e.g. quetiapine), which are identified as high risk of misuse, diversion and dependence in prison by the UK Royal College of General Practitioners Safer Prescribing in Prisons (2019) guidelines [22]. Future work is needed to examine prescribing trends for these drugs in Irish prisons.

Notwithstanding these limitations, this is the first study to examine prescribing trends for opioids, benzodiazepines, Z-drugs and gabapentinoids in Irish prisons in recent years. This is important, as up-to-date, robust evidence is necessary to inform practices and policies. In addition, while other studies typically used census data [18, 56], this work reports on electronic prescribing record data from all Irish prisons from 2012 to 2020, providing national estimates of prescribing rates and long-term trends. All prescribing in prison being electronically recorded, missing data are unlikely. Moreover, given the great imbalance in gender ratio observed in prison, it is critical to run gender sensitive analyses, as women specific results would otherwise remain invisible. Finally, history of OUD was taken into account in analyses and appears as a highly relevant factor for prescribing patterns in prison.

## Conclusion

This study examined prescribing trends in Irish prisons for opioids, benzodiazepines, Z-drugs and gabapentinoids from 2012 to 2020. Opioid prescribing rates were halved between 2015 and 2020 and benzodiazepine and Z-drug prescribing rates followed downward trends, which is in line with recent guidelines for safe prescribing in prisons [22]. In contrast, gabapentinoid prescribing increased over the study period (although appears to be reducing in 2020), largely driven by pregabalin. This is a significant concern given the increased risk of drug-related poisoning [42]. Analyses by gender identified a higher prevalence of OUD, as well as benzodiazepines and Z-drugs prescribing among women in prison compared to men, underlining the burden of addiction/mental health issues and specific needs in this population. A history of OUD was associated with increased prescribing rates in men, and appears as a relevant flag for targeted interventions. In a context of high risk for misuse, balancing the benefits and risks of prescribing drugs in prison is complex, calling for more published evidence on current healthcare practices and the needs of people in prison.

### Supplementary Information

**Additional file 1.**

## Data Availability

The datasets analysed in this article were provided to the research team by the data controller (Irish prison service) for the sole purpose of this study under a data sharing agreement and cannot be shared with third parties. Dr. Gráinne Cousins can be contacted in relation to data queries.
