# Supplementary material for: Prescription drugs with potential for misuse in Irish prisons: analysis of national prison prescribing trends, by gender and history of opioid use disorder, 2012 to 2020
Source: BMC Psychiatry. 2023 Oct 6;23:725. doi: 10.1186/s12888-023-05195-9 (PMC10559424; doi:10.1186/s12888-023-05195-9)
Supplement: Supplementary file 1 — Additional file 1. [file 12888_2023_5195_MOESM1_ESM.zip › 12888_2023_5195_MOESM2_ESM.docx]

**Additional files:**


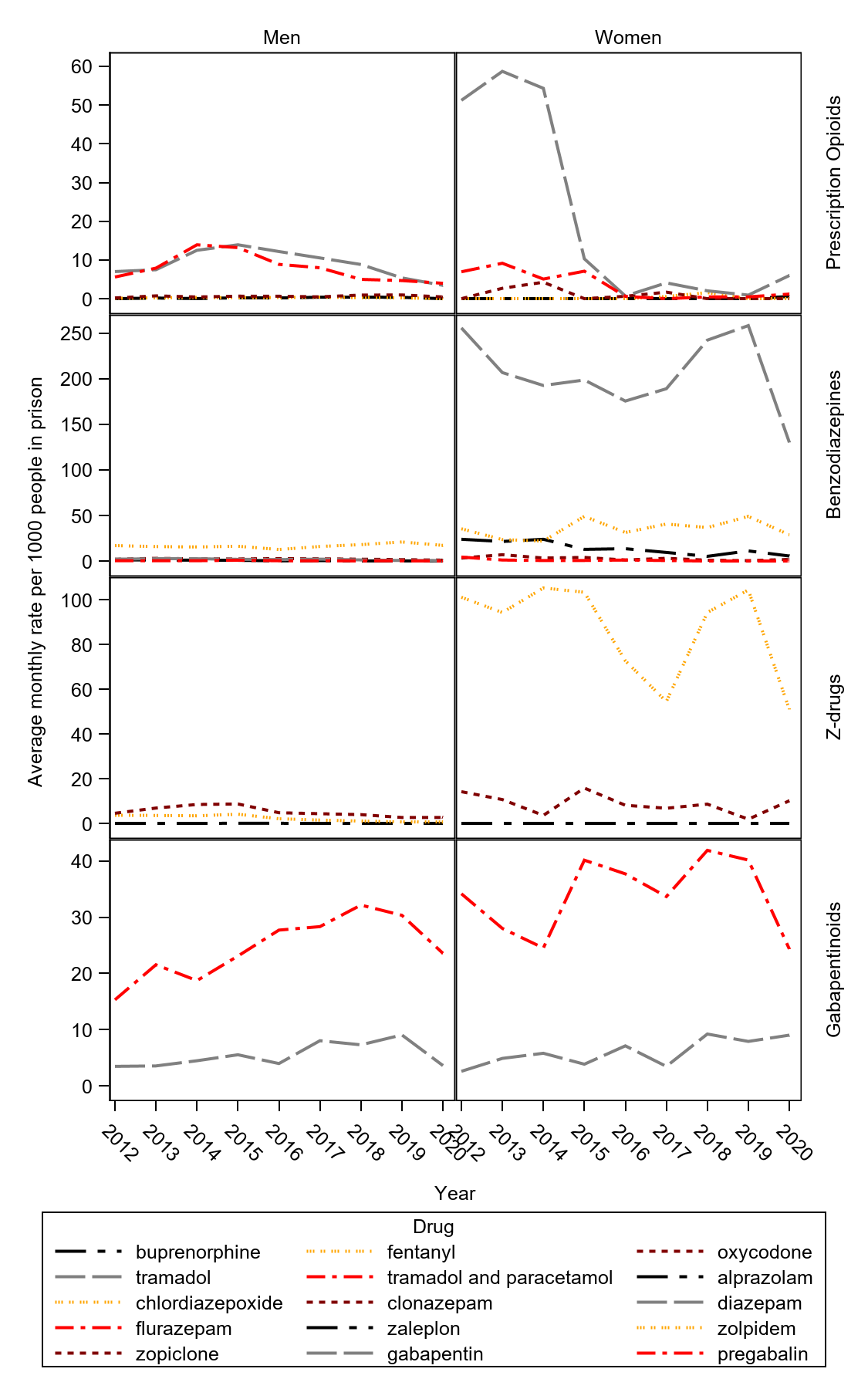


Additional figure: Annual average prescribing rates of opioids, benzodiazepines, Z-drugs and gabapentinoids, by drug (ATC code)
